# Supplementary material for: Artificial Intelligence-Assisted Loop Mediated Isothermal Amplification (AI-LAMP) for Rapid Detection of SARS-CoV-2
Source: Viruses. 2020 Sep 1;12(9):972. doi: 10.3390/v12090972 (PMC7552048; doi:10.3390/v12090972)
Supplement: Supplementary file 1 [file viruses-12-00972-s001.zip › viruses-885786-suppproof/viruses-885786-suppconv.pdf]

viruses

MDPI

Supplementary Materials: Artificial Intelligence-Assisted Loop Mediated Isothermal Amplification (ai-LAMP) for Rapid Detection of SARS-CoV-2

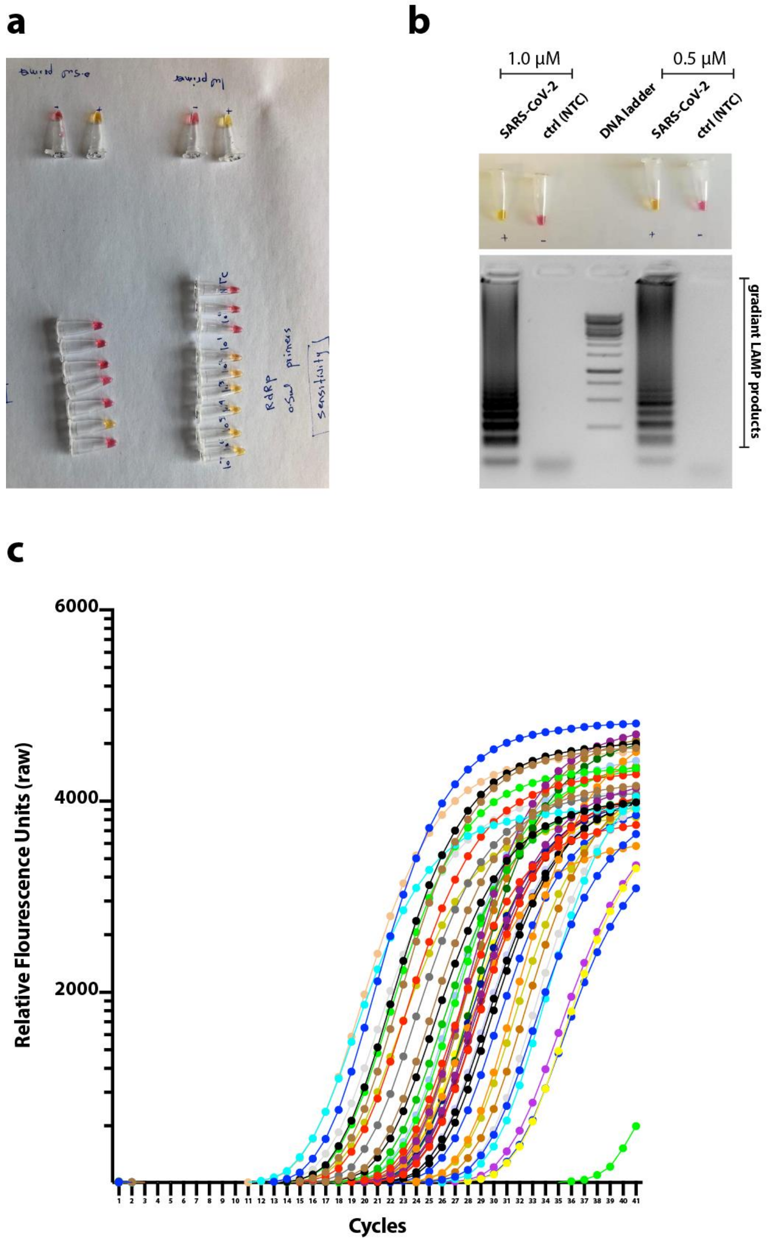

Figure 1. Optimization of LAMP for the detection of SARS-CoV-2. (**a**) Sensitivity and specificity of the LAMP primers used in the study pertaining to the Fig. 2 and 3 in the main manuscript. (**b**) Optimization of different primers concentration used in the ai-LAMP assay. (**c**) Raw fluorescence units for the spiked miRNA detected by the qRT-LAMP. Associated Ct values are provided in the Fig. 6H in the main manuscript.
